# Predictors of bacteremia in febrile infants under 3 months old in the pediatric emergency department

**DOI:** 10.1186/s12887-023-04271-z

**Published:** 2023-09-07

**Authors:** Bei-Cyuan Guo, Yin-Ting Chen, Yu-Jun Chang, Chun-Yu Chen, Wen-Ya Lin, Han-Ping Wu

**Affiliations:** 1 Department of Pediatrics, College of Medicine, National Cheng Kung University Hospital, National Cheng Kung University, Tainan, Taiwan; 2 Division of Neonatology, Department of Pediatrics, Children Hospital, China Medical University, Taichung, Taiwan; 3 Laboratory of Epidemiology and Biostastics, Changhua Christian Hospital, Changhua, Taiwan; 4 Department of Emergency Medicine, Tungs’ Taichung MetroHarbor Hospital, Taichung, Taiwan; 5 Department of Pediatric Emergency Medicine, Department of Pediatrics, Taichung Veteran General Hospital, Taichung, Taiwan; 6 College of Medicine, Chang Gung University, Taoyuan, Taiwan; 7 Department of Pediatrics, Chiayi Chang Gung Memorial Hospital, No. 6, W. Sec., Jiapu Rd, Puzi City, Taiwan

**Keywords:** Infant, Fever, Bacteremia, Emergency department

## Abstract

**Introduction:**

Fever may serve as the primary indicator of underlying infection in children admitted to the pediatric emergency department (PED), especially in high-risk young infants. This study aimed to identify early clinical factors that could help predict bacteremia in young febrile infants.

**Methods:**

The study included infants under 90 days of age who were admitted to the PED due to fever. Patients were divided into two groups based on the presence or absence of bacteremia and further divided into three age groups: (1) less than 30 days, (2) 30 to 59 days, and (3) 60 to 90 days. Several clinical and laboratory variables were analyzed, and logistic regression and receiver operating characteristic (ROC) analyses were used to identify potential risk factors associated with bacteremia in young febrile infants.

**Results:**

A total of 498 febrile infants were included, of whom 6.4% were diagnosed with bacteremia. The bacteremia group had a higher body temperature (BT) at triage, especially in neonates, higher pulse rates at triage, longer fever subsidence time, longer hospital stays, higher neutrophil counts, and higher C-reactive protein (CRP) levels than those of the non-bacteremia group. ROC analysis showed that the best cut-off values for predicting bacteremia in infants with pyrexia were a BT of 38.7 °C, neutrophil count of 57.9%, and CRP concentration of 53.8 mg/L.

**Conclusions:**

A higher BT at triage, increased total neutrophil count, and elevated CRP levels may be useful for identifying bacteremia in young febrile infants admitted to the PED.

## Introduction

Fever often serves as the primary indicator of an underlying infection in neonates and young infants, prompting caregivers to seek immediate medical attention at a pediatric emergency department (PED) [1, 2]. Although viral infections are the most common cause of fever in young infants, some may experience fever without clear signs or symptoms, leading to a diagnosis of fever without an identifiable cause based on the initial clinical assessment [3, 4]. Severe bacterial infections, such as bacteremia, can also occur in young infants.

Bacteremia is a medical condition characterized by the presence of bacteria in the bloodstream, typically resulting from an infection or disease, and can lead to a more critical condition known as septicemia [5, 6] Early identification of bacteremia in young infants is crucial; however, when assessing febrile infants, primary clinicians often face challenges in predicting bacteremia based solely on clinical presentation in the absence of blood culture findings [7, 8].As a result, it is crucial for primary physicians in the PED to identify the risk factors associated with bacteremia in young febrile infants who are at high risk of bacteremia and require immediate hospitalization for empiric antimicrobial therapy and intensive care. In this study, we aimed to analyze the data of febrile infants under 90 days of age admitted to the PED to understand the initial presentations and clinical characteristics of young febrile infants with bacteremia.

## Materials and methods

### Patient population and study design

This retrospective cohort study included febrile young infants under 90 days of age who were admitted to the PED of a medical center between 2014 and 2017. Infants with premature birth, immunodeficiency diseases, any chronic diseases requiring antibiotic prophylaxis, or those who had received immunizations within 48 h prior to the PED visit were excluded from the analysis. The patient did not take any antipyretic dugs before arriving at the PED. Patients were categorized into three groups based on age: (1) less than 30 days, (2) 30 to 59 days, and (3) 60 to 89 days. This study was approved by the Institutional Review Board and Ethics Committee of China Medical University Hospital (CMUH108-REC1-061), and all methods were performed in accordance with relevant guidelines and regulations. Data were collected, reviewed, de-identified, and anonymized prior to the analysis. The Institutional Review Board and Ethics Committee of China Medical University Hospital waived the requirement for informed consent due to the anonymized nature of the data and the scientific purpose of the study.

### Measurements

Fever was defined as a documented body temperature (BT) greater than 38.0 °C, obtained either at home or in the PED through any temperature measurement method (e.g., rectal, axillary). Infants with fever were divided into two groups: those with bacteremia and those without. Bacteremia was defined as a positive blood culture result for a pathogen. The study collected and analyzed the following variables: age, sex, gestational age (GA), delivery method, birth weight, body weight at the PED, vital signs (including body temperature, pulse rate, respiratory rate, and blood pressure) which were taken during triage at the point of visiting the ED, length of hospital stay, and laboratory test results at admission. Fever subsidence time is defined as the duration in days from the first body temperature above 38 °C to the time when the temperature fell below 38 °C for the first time and no fever episode is detected for a consecutive 48-hour period, regardless of taking antipyretic drugs such as acetaminophen syrup. Laboratory tests performed at admission included total white blood cell (WBC) count, neutrophil count, non-segmented bands, and C-reactive protein (CRP) levels. All clinical and laboratory variables were compared between the bacteremia and non-bacteremia groups based on the different age groups to identify the relevant risk factors for bacteremia in young febrile infants.

### Statistical analysis

Statistical analyses were performed using the t-test, Mann-Whitney U test, Fisher’s exact test, chi-square test, and logistic regression analyses. Logistic regression analyses were performed to evaluate the influences of various factors on blood culture growth. Receiver operating characteristic (ROC) analysis was used to identify the ideal cutoff values for the clinical variables of interest, which were assessed based on sensitivity, specificity, area under the ROC curve, positive likelihood ratio (LR+), and negative likelihood ratio (LR-). In the descriptive analysis, percentages, or medians (first and third quartiles) were used to present values. Differences between groups are presented as 95% confidence intervals. Statistical significance was defined as P < 0.05, and all statistical analyses were conducted using the IBM SPSS Statistics software (version 22.0; SPSS Inc., Chicago, IL, USA).

## Results

A total of 498 young febrile infants admitted to the PED during the study period were enrolled in this study. All patients had undergone blood tests and blood culture test. The infants had a median age of 54 days (interquartile range, IQR, 33 days – 72 days), with a male-to-female ratio of 1.91. Of the total cases, 6.0% had a definite diagnosis of bacteremia (Table 1). No significant differences were found in age, sex, GA, birth weight, or admission weight between the bacteremia and non-bacteremia groups. However, the bacteremia group displayed higher BT at triage, higher pulse rates at triage, and the highest BT during the hospital stay (all P < 0.05). The bacteremia group exhibited a longer fever subsidence time compared to the non-bacteremia group (3.0 vs. 2.0 days, P < 0.001), as well as a longer hospital stay (15.0 vs. 4.0 days, P < 0.001). In laboratory examinations, the percentage of neutrophils and CRP levels were significantly higher in the bacteremia group than in the non-bacteremia group (both P < 0.05). However, there were no significant differences in total WBC counts and bands. Logistic regression analysis showed that higher BT at triage, higher pulse rates at triage, higher neutrophil counts, and higher CRP levels were all associated with increased odds of bacteremia. Furthermore, the logistic regression analysis revealed that higher body temperature at triage, elevated pulse rates at triage, increased neutrophil counts, and higher CRP levels were all associated with an increased likelihood of bacteremia. After adjusting for other covariates, age between 30 and 59 days, higher BT at triage, higher neutrophil count, and higher CRP levels were associated with bacteremia (Table 2). The risk of bacteremia in infants aged between 30 and 59 days was 3.148 times higher than in infants aged between 60 and 89 days (95% CI: 1.199–8.266, P = 0.020). Additionally, a higher body temperature at triage was found to be associated with a greater risk of bacteremia (Odds ratio = 1.871, 95% CI = 1.107–3.162, P = 0.020). Specifically, for each 1-degree Celsius increase in body temperature at triage, the risk of bacteremia in newborns increased by 87.1%. Moreover, higher levels of both neutrophils and CRP were also associated with an increased risk of bacteremia in newborns. For each additional unit (1%) increase in neutrophils, the risk of bacterial infection in newborns increased by 6.6%, while for each additional unit (1 mg/L) increase in CRP, the risk of bacterial infection in newborns increased by 9.4%.

**Table 1:** Comparison of clinical characteristics, vital signs, and blood laboratory findings in febrile infants in the Bacteremia and Non-bacteremia groups

	Number (%) or Median (Q1, Q3)		
Variables	Bacteremia(n = 32)	Non-bacteremia (n = 466)	*P*-value
Age(d)	50.0(27.5, 58.5)	55.5(33.0, 72.0)	0.129
Male sex, %	20(62.5%)	307(65.9%)	0.697
GA(wk)	38.5(37.5, 39.5)	38.0(37.0, 39.0)	0.746
Birth weight (g)	3070.0(2850.0, 3167.5)	3082.0(2800.0, 3340.0)	0.240
Admission weight (g)	4837.5(4082.5, 5290.0)	4897.5(3995.0, 5590.0)	0.439
BT at triage (°C)	38.70(38.55, 39.20)	38.20(37.70, 38.70)	< 0.001
Pulse rates at triage (/min)	185.0(180.0, 200.0)	168.0(156.0, 184.0)	< 0.001
Highest BT(°C)	39.15(38.70, 39.50)	38.50(38.00, 39.00)	< 0.001
Highest BT of hospital stay (°C)	38.80(38.20, 39.40) (n = 26)^*^	38.50(38.10, 38.90) (n = 205)^*^	0.013
Fever subsidence time (d)†	3.0(2.0, 4.0)	2.0(1.0, 3.0)	0.001
Duration of hospitalization(days)	15.0(13.0, 17.0)	4.0(3.0, 5.0)	< 0.001
Laboratory test (blood)			
WBC(/uL)	10850.0(7150.0, 14100.0)	11500.0(8100.0, 15800.0)	0.314
Neutrophils (%)	62.15(56.75, 68.30)	47.60(36.60, 57.40)	< 0.001
Bands (%)	0.0(0.0, 0.0)	0.0(0.0, 0.0)	0.883
CRP (mg/L)	24.7(3.7, 77.2)	8.9(1.9, 32.8)	0.003

BT = body temperature, CRP = C-reactive protein, GA = gestational age, WBC = white blood cell

∗Data collected in patients who still had a body temperature of > 38 °C after admission

† Defined as the days during which no fever episode was detected for a consecutive 48-h period

**Table 2:** Logistic regression analysis of factors associated with bacteremia

		Univariate analysis				Multivariate analysis (adjusted)		
Variables	N (%) or Median(Q1, Q3)	OR	95% C.I.	P-value		OR	95% C.I.	P-value
Gender								
female	12(7.0%)	1.158	0.552–2.430	0.697				
male	20(6.1%)	1.000						
Age(day)	50.0(27.5, 58.5)	0.989	0.975–1.004	0.152				
< 30	11(9.0%)	2.552	0.997–6.529	0.051		2.523	0.924–6.891	0.071
30 ~ 59	13(8.0%)	2.247	0.908–5.557	0.080		3.148	1.199–8.266	0.020
60–89	8(3.7%)	1.000				1.000		
Admission weight(kg)	4.84(4.08, 5.29)	0.874	0.631–1.210	0.417				
BT at triage (°C)	38.7(38.6, 39.2)	2.485	1.545–3.997	< 0.001		1.871	1.107–3.162	0.019
Highest BT(°C)	38.8(38.2, 39.4)	3.041	1.839–5.028	< 0.001				
Pulse rates at triage (/min)	185(180, 200)	1.045	1.026–1.065	< 0.001				
Laboratory test (blood)								
WBC(/uL)/100	10.9(7.2, 14.1)	0.978	0.918–1.042	0.493				
Neutrophils (%)	62.2(56.8, 68.3)	1.072	1.041–1.104	< 0.001		1.066	1.033–1.101	< 0.001
CRP (mg/L)	24.7(3.7, 77.2)	1.100	1.041–1.163	0.001		1.094	1.024–1.170	0.008

BT = body temperature, CRP = C-reactive protein, WBC = white blood cell, OR: odds ratio

Infants with bacteremia exhibited varying clinical characteristics depending on their age group. In all three age groups, infants with bacteremia had higher pulse rates and longer hospital stays (all P < 0.05). In age groups 1 and 3, infants with bacteremia had a higher BT at triage (both P < 0.05), while a longer fever subsidence time was only observed in age group 1. In laboratory examinations, higher neutrophil counts were found in infants with bacteremia in all three age groups, but higher CRP levels were only noted in age groups 1 and 2 (both P < 0.05) (Table 3). Furthermore, multivariable analysis analyses were performed to identify factors associated with bacteremia in the different age groups, including young infants below 90 days of age, as shown in Table 4. After adjusting for other covariates, infants younger than 30 days with a higher BT at triage were found to have increased odds of developing bacteremia. In infants aged 30–59 and 60–90 days, higher neutrophil and CRP levels were associated with bacteremia. Additionally, female infants aged 60–89 days presented a higher incidence of bacteremia.

**Table 3:** Clinical findings and laboratory results among various age groups in young infants below 90 days of age in the Bacteremia and Non-bacteremia groups

	N(%) or Median(Q1, Q3)										
	< 30d (n = 122)				30-59d(n = 162)				60-89d(n = 214)		
**Variables**	Bacteremia	Non-bacteremia	***P*** **-value**		Bacteremia	Non-bacteremia	***P*** **-value**		Bacteremia	Non-bacteremia	***P*** **-value**
Male sex, %	7(63.6%)	70(63.1%)	> 0.999		11(84.6%)	97(65.1%)	0.223		2(25.0%)	140(68.0%)	0.019
GA(wk)	39.0(38.0, 40.0)	38.0(38.0, 40.0)	0.560		39.0(38.0, 39.0)	38.0(38.0, 39.0)	0.706		37.5(36.5, 39.5)	38.0(37.0, 39.0)	0.448
Birth weight (g)	2980.0(2850.0, 3275.0)	3150.0(2840.0, 3425.0)	0.433		3070.0(2980.0, 3150.0)	3037.5(2750.0, 3300.0)	0.747		3105.0(2632.5, 3120.0)	3092.5(2845.0, 3355.0)	0.316
Admission weight (g)	4065.0(3385.0, 4550.0)	3495.0(3045.0, 3905.0)	0.032		4800.0(4300.0, 4990.0)	4600.0(4245.0, 5060.0)	0.702		5290.0(5102.5, 5420.0)	5632.0(5127.5.0, 6035.0)	0.203
BT at triage (°C)	39.20(38.60–39.50)	38.20(37.90, 38.70)	< 0.001		38.70(38.00, 38.70)	38.20(37.90, 38.70)	0.240		38.95(38.70, 39.60)	38.40(37.70, 39.00)	0.014
Pulse rates at triage (/min)	180.0(173.0-186.0)	163.0(153.0, 182.0)	0.010		190.0(180.0, 204.0)	168.0(156.0, 185.0)	< 0.001		182.0(182.0, 215.0)	170.0(157.0, 185.0)	0.004
Highest BT(°C)	39.20(38.80, 39.50)	38.30(38.00, 38.70)	< 0.001		39.10(38.70, 39.50)	38.40(38.00, 38.90)	0.018		39.05(38.75, 39.65)	38.60(38.00, 39.10)	0.057
Highest BT of hospital stay (°C)	38.70(38.20, 39.20)	38.40(38.10, 38.60)	0.053		39.10(39.00, 39.50)	38.50(38.10, 38.90)	0.004		38.40(38.20, 38.60)	38.60(38.10, 39.00)	0.931
Fever subsidence time (d)∗	3.0(2.0, 3.0)	1.0(1.0, 3.0)	0.002		2.5(1.5, 5.5)	2.0(1.0, 3.0)	0.078		2.0(2.0, 3.0)	2.0(1.0, 3.0)	0.247
Duration of hospitalization(days)	15.0(11.0, 16.0)	4.0(3.0, 7.0)	< 0.001		16.0(15.0, 17.0)	4.0(3.0, 6.0)	< 0.001		15.0(11.0, 19.5)	4.0(3.0, 5.0)	< 0.001
Laboratory test (blood)											
WBC(/uL)	9700.0(4400.0, 12100.0)	12000.0(9300.0, 15700.0)	0.241		11500.0(7900.0, 14100.0)	9500.0(7000.0, 13500.0)	0.767		12150.0(7750.0, 15550.0)	13000.0(9300.0, 17100.0)	0.576
Neutrophils (%)	60.10(55.20, 65.00)	50.00(41.40, 61.00)	0.032		64.50(59.30, 72.59)	45.70(33.00, 53.70)	< 0.001		64.50(56.75, 68.80)	47.90(37.79, 58.00)	0.005
Bands (%)	0.0(0.0, 0.0)	0.0(0.0, 0.0)	0.393		0.0(0.0, 0.0)	0.0(0.0, 0.0)	0.551		0.0(0.0, 0.0)	0.0(0.0, 0.0)	0.121
CRP (mg/L)	39.6(3.0, 78.7)	4.9(0.6, 32.3)	0.043		27.8(4.8, 121.4)	0.67(0.12, 2.75)	0.012		21.5(1.7, 68.3)	11.3(3.0, 38.1)	0.568

BT = body temperature, CRP = C-reactive protein, GA = gestational age, WBC = white blood cell

∗Defined as the date during which no fever episode was detected for a consecutive 48-h period

**Table 4:** Multiple logistic regression to identify factors associated with bacteremia among various age groups in young infants below 90 days of age

	Age < 30d				Age 30-59d				Age 60-89d		
Variables	OR	95% C.I.	P-value		OR	95% C.I.	P-value		OR	95% C.I.	P-value
Gender (F vs. M)	1.171	0.391–7.578	0.473		0.392	0.077–2.006	0.261		9,621	1.445–64.052	0.019
BT at triage(°C)	6.134	1.870-20.137	0.003		0.950	0.448–2.015	0.873		1.693	0.583–4.920	0.333
Neutrophils (%)	1.309	0.983–1.099	0.179		1.080	1.024–1.138	0.004		1.084	1.004–1.170	0.040
CRP (mg/L)	1.023	0.917–1.141	0.684		1.205	1.033–1.405	0.018		1.193	1.016–1.401	0.031

BT = body temperature, CRP = C-reactive protein, OR: odds ratio

In classification problems, the AUC-ROC curve serves as a performance metric across different threshold settings. The ROC (Receiver Operating Characteristic) is a graphical representation of the classifier’s performance at various thresholds, and the AUC (Area Under the Curve) quantifies the degree of separability between classes. The results of the ROC analysis for predicting bacteremia in young febrile infants are shown in Table 5. The following criteria had a sensitivity of 100% for excluding pediatric bacteremia: BT ≤ 37.7 °C, neutrophil count ≤ 6.0%, and CRP levels ≤ 1.15 mg/L. On the other hand, the following criteria had a specificity of 100% for detecting the condition: BT ≥ 42.1° C, neutrophil count ≥ 83.0%, and CRP levels ≥ 336.5 mg/L. Table 6 shows the diagnostic accuracies for predicting acute bacteremia of the parameters at different cutoff values. A BT at triage of ≥ 38.7 °C exhibited a sensitivity of 71.9% and a specificity of 67.0%, with an AUC of 0.710. The highest BT (≥ 38.7 °C) had a sensitivity of 84.4% and a specificity of 59.5%, with an AUC of 0.745. A pulse rate of ≥ 170 bpm at triage demonstrated a sensitivity of 96.8% and a specificity of 52.4%, with an AUC of 0.769. Neutrophil percentages of ≥ 57.9% had a sensitivity of 75.0% and a specificity of 76.1%, with an AUC of 0.773. Lastly, CRP levels of ≥ 53.8 mg/L exhibited a sensitivity of 43.8% and a specificity of 86.2%, with an AUC of 0.657. Among infants with bacteremia, Group B Streptococcus (GBS) and Escherichia coli (E. coli) were the most frequently identified pathogens, with 16 and 12 cases, respectively. GBS was the leading cause of bacteremia during the second month of life.

**Table 5:** Cutoff value in sensitivity = 1.0 or specificity = 1.0 for the prediction of bacteremia

Variables	Cutoff	Sensitivity	Specificity	PPV	NPV	+LR	-LR
Body temperature(°C)	37.7	1.000	0.189	0.078	1.000	1.233	0.000
	42.1	0.000	1.000	-	0.936	-	1.000
Neutrophils (%)	6.0	1.000	0.002	0.064	1.000	1.002	0.000
	83.0	0.000	1.000	-	0.936	-	1.000
CRP (mg/L)	1.15	1.000	0.198	0.079	1.000	1.247	0.000
	336.5	0.000	1.000	-	0.936	-	1.000

CRP = C-reactive protein, PPV = Positive predictive value, NPV = Negative predictive value, LR: likelihood ratio

**Table 6:** Receiver operating characteristic curve analysis of factors for the prediction of bacteremia

	Criterion values and coordinates of ROC curve								Area under the ROC curve					
Variables	Value	Sensitivity	Specificity	PPV	NPV	+LR	-LR		Area	SE	95% C.I.			P-value
BT at triage(°C)	>=38.7	0.719	0.670	0.130	0.972	2.180	0.420		0.710	0.042	0.627	-	0.793	< 0.001
Highest BT(°C)	>=38.7	0.844	0.595	0.125	0.982	2.085	0.263		0.745	0.042	0.663	-	0.826	< 0.001
Pulse rates at triage (/min)	>=170	0.968	0.524	0.122	0.996	2.031	0.062		0.769	0.032	0.707	-	0.831	< 0.001
Neutrophils (%)	>=57.9	0.750	0.761	0.177	0.978	3.142	0.328		0.773	0.041	0.693	-	0.853	< 0.001
CRP (mg/L)	>=53.8	0.438	0.862	0.179	0.957	3.172	0.653		0.657	0.052	0.556	-	0.758	0.003

BT = body temperature, CRP = C-reactive protein, LR: likelihood ratio

## Discussion

Pyrexia is a common indicator of infection in neonates and young infants, and early identification of bacteremia is crucial for timely treatment and favorable outcomes. Of note, vital signs are important parameters for determining the physical status of children. Previous studies have suggested that a high BT in febrile young infants visiting the PED may be a risk factor for bacteremia [9]. In our study, we also found that febrile young infants with bacteremia had a higher BT, especially those aged less than 30 days and between 60 and 89 days. Additionally, the highest BT in infants with bacteremia was higher than that in infants without bacteremia, particularly in infants aged less than 60 days. Our results showed that both BT at triage ≥ 38.7 °C and the highest recorded BT ≥ 38.7 °C had an AUC greater than 0.7, indicating a risk for bacteremia. Clinically, if the BT was greater than 38.7 °C in infants with fever admitted to the PED, the probability of bacteremia increased.

In previous studies, it was observed that newborns with bacteremia had significantly higher maximum mean heart rates [10]. In our study, we found that febrile young infants under 90 days of age had higher pulse rates at triage than those of infants without bacteremia. The AUC for pulse rates ≥ 170 bpm at triage was also greater than 0.7. However, pulse rates may be correlated with BT; therefore, BT at triage should be considered a confounding factor, and the role of pulse rates in predicting bacteremia should be adjusted in this population.

Our findings did not show any significant differences in WBC counts between infants with and without bacteremia. Prior studies have also indicated that routine blood tests, such as WBC counts or absolute neutrophil counts, are not a reliable method for predicting bacteremia in younger febrile infants [9, 11]. However, we observed a higher percentage of neutrophils in infants with bacteremia than in those without. Based on the results of the multiple logistic regression analysis, we found that higher percentages of neutrophils in infants aged 30–89 days were associated with a high risk of bacteremia. Moreover, infants with bacteremia, particularly those aged less than 60 days, had significantly higher CRP levels than those without bacteremia. Previous studies have also reported that CRP levels may be an indicator of bacteremia in young infants and children [12, 13]. Therefore, we believe that CRP levels could serve as a useful predictor of bacteremia in young febrile infants. Based on the ROC analysis results, we established significant cutoff values for neutrophil counts and CRP concentration to discriminate the presence or absence of bacteremia in this population. Our findings revealed that the probability of bacteremia increased when the neutrophil count was higher than 83% and the CRP levels were greater than 336.5 mg/L. Other biomarkers, such as procalcitonin or presepsin (sCD14-ST), could be considered for the detection of serious bacterial infection. A previous study demonstrated the favorable test characteristics of procalcitonin in identifying bacteremia in young febrile infants under 90 days of age [14]. Moreover, other studies have suggested that procalcitonin is a reliable marker for identifying or ruling out invasive bacterial infections in febrile infants less than 3 months [15, 16]. Additionally, presepsin has exhibited a sensitivity of over 90% for sepsis, with a critical threshold at approximately 650 ng/L [17, 18]. Investigations into its application for the early diagnosis of sepsis in pediatric emergency room settings have been undertaken [18]. Furthermore, presepsin is regarded as a potential marker of sepsis in neonates due to its limited susceptibility to variables that commonly influence CRP and PCT levels in these subjects [19].While Procalcitonin and presepsin show promise as valuable predictors for bacteremia, further research is required to fully establish their potential in clinical practice. Another observation from the study was that female infants aged 60–89 days had a higher incidence of bacteremia. In previous studies, one research reported that females accounted for 36% of bacteremia cases in the age group between 1 week to 3 months [8], while another study found that 49% of febrile infants aged ≤ 90 days with bacteremia were female [20].Additionally, other reports revealed that 35% of bacteremia patients were female in infants less than one year [21]. This discrepancy in findings warrants further investigation to better understand the underlying factors contributing to the higher incidence of bacteremia in this specific population of female infants.

Clinically, tests for diagnosing bacteremia are often classified into three diagnostic test zones: a high-sensitivity zone, a high-specificity zone, and an indeterminate zone. Based on our findings, we identified two cutoff points for each variable that could be more easily applied in clinical practice to rule in or rule out bacteremia in infants with fever who are admitted to the PED. We found that serum parameters were highly valid for diagnosing bacteremia when neutrophil counts were ≥ 83% or CRP levels were ≥ 336.5 mg/L in febrile infants. Conversely, for ruling out bacteremia, neutrophil counts of 6% or lower on day 1 and CRP levels of 1.15 mg/L or lower on day 2 and of 4.6 mg/L or lower on day 3 were found to be effective. Additionally, infants were more likely to have bacteremia when their BT was greater than 42.1 °C, while bacteremia could be ruled out when their BT was less than 37.7 °C.

Previous studies have indicated a reduction in the incidence of bacteremia, which has been attributed to expanded vaccine coverage, maternal GBS screening, and increased use of intrapartum antibiotics [22, 23]. Our study revealed an overall bacteremia rate of approximately 6.02%, which is higher than the previously reported rate of approximately 2% for febrile infants who underwent blood culture testing [9, 24]. However, we observed a declining trend in the prevalence of bacteremia, with rates decreasing from 9.01% in the neonatal group to 8.02% in infants aged 30–59 days, and finally to 2.8% in infants aged 60–89 days. Other studies have also reported a generally higher prevalence of bacteremia within the first four weeks of life [24, 25].

This study had certain limitations that should be acknowledged. First, due to its retrospective design, it was challenging to establish causal relationships between the risk factors and bacteremia. Second, the study was conducted in a single medical center in Taiwan, which may limit the generalizability of the findings. Finally, certain potential risk factors for bacteremia, such as the duration of fever before presenting to the PED, were not included in the analysis.

## Conclusion

Clinical presentation, including BT at triage, highest BT, pulse rate at triage, and blood laboratory parameters, may serve as valuable indicators of bacteremia in infants. Higher BT at triage, increased neutrophil counts, and elevated CRP levels could be helpful clinical factors for predicting the risk of bacteremia in young febrile infants with pyrexia admitted to the PED.

## Data Availability

The datasets used and/or analyzed during the current study are available from the corresponding author upon reasonable request.
